# Pro-inflammatory Diet Pictured in Children With Atopic Dermatitis or Food Allergy: Nutritional Data of the LiNA Cohort

**DOI:** 10.3389/fnut.2022.868872

**Published:** 2022-04-08

**Authors:** Olivia Schütte, Larissa Bachmann, Nitin Shivappa, James R. Hebert, Janine F. Felix, Stefan Röder, Ulrich Sack, Michael Borte, Wieland Kiess, Ana C. Zenclussen, Gabriele I. Stangl, Gunda Herberth, Kristin M. Junge

**Affiliations:** ^1^Department of Environmental Immunology, Helmholtz Centre for Environmental Research – UFZ, Leipzig, Germany; ^2^Cancer Prevention and Control Program, University of South Carolina, Columbia, SC, United States; ^3^Department of Epidemiology and Biostatistics, Arnold School of Public Health, University of South Carolina, Columbia, SC, United States; ^4^Department of Nutrition, Connecting Health Innovations LLC, Columbia, SC, United States; ^5^The Generation R Study Group (Na-2918), Erasmus MC, University Medical Centre Rotterdam, Rotterdam, Netherlands; ^6^Department of Pediatrics, Erasmus MC, University Medical Centre Rotterdam, Rotterdam, Netherlands; ^7^Institute of Clinical Immunology, Medical Faculty, University of Leipzig, Leipzig, Germany; ^8^Children’s Hospital, Municipal Hospital “St. Georg,” Leipzig, Germany; ^9^Center for Pediatric Research Leipzig, Medical Faculty, University Hospital for Children and Adolescents, University of Leipzig, Leipzig, Germany; ^10^Perinatal Immunology, Medical Faculty, Saxonian Incubator for Clinical Translation (SIKT), University of Leipzig, Leipzig, Germany; ^11^Institute for Agricultural and Nutritional Science, Martin Luther University Halle-Wittenberg, Halle, Germany

**Keywords:** atopic dermatitis (AD), food allergy (FA), food frequency questionnaires (FFQ), nutrients, food group consumption, C-DII, 10-year-old children

## Abstract

**Background:**

Lifestyle and environmental factors are known to contribute to allergic disease development, especially very early in life. However, the link between diet composition and allergic outcomes remains unclear.

**Methods:**

In the present population-based cohort study we evaluated the dietary intake of 10-year-old children and analyses were performed with particular focus on atopic dermatitis or food allergy, allergic diseases known to be affected by dietary allergens. Dietary intake was assessed *via* semi-quantitative food frequency questionnaires. Based on these data, individual nutrient intake as well as children’s Dietary Inflammatory Index (C-DII™) scores were calculated. Information about atopic manifestations during the first 10 years of life and confounding factors were obtained from standardized questionnaires during pregnancy and annually thereafter.

**Results:**

Analyses from confounder-adjusted logistic regression models (*n* = 211) revealed that having atopic outcomes was associated with having a pro-inflammatory pattern at the age of 10 years: OR = 2.22 (95% CI: 1.14–4.31) for children with atopic dermatitis and OR = 3.82 (95% CI: 1.47–9.93) for children with food allergy in the first 10 years of life.

**Conclusion:**

A pro-inflammatory dietary pattern might worsen the atopic outcome and reduce the buffering capacity of the individual against harmful environmental exposures or triggers. For pediatricians it is recommended to test for the individual tolerance of allergenic foods and to increase the nutrient density of tolerable food items to avoid undesirable effects of eating a pro-inflammatory diet.

## Graphical Abstract

Aim and design of the analysed project within the prospective LiNA cohort.

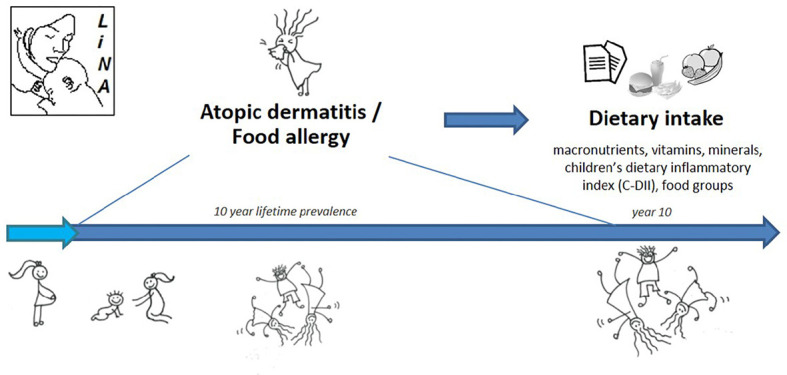

## Introduction

In Western countries the increasing prevalence of atopic diseases has become a major problem in human health. Because allergy onset and atopic march begin in infancy (1), early prevention is advisable. Worldwide, approximately 15%–30% of children live with dermatitis (2, 3) and 4–10% of children suffer from food allergy (4–6). Both atopic dermatitis and food allergy have the earliest onset within the atopic march, resulting in highest prevalence in children before school age (7). Many risk factors for the development of atopic diseases – acting independently or in multifactorial combination – have been identified (8, 9). These include genetic background, individual immune response, barrier dysfunctions, microbiome alterations, as well as lifestyle behaviors (10, 11) and environmental conditions (12).

Diet represents a source of components that could affect atopy in a number of ways. First, diet is a potential source of allergens. Diet also could provide substrate for components that interfere with the pathology of atopy. Finally, it is well-known that diet can modulate inflammatory and related immune responses that can ameliorate or exacerbate allergic or atopic reactions. However, the link between diet composition and the pathogenesis of allergies is complex and not well understood. Though the important role of breastfeeding and timing/manner of introducing solid food is well understood, there are few dietary factors consumed in early life that are described to alter the risk for allergic diseases [e.g., vitamin D, pro/prebiotics or omega(ω)-3 long-chain polyunsaturated fatty acids (13, 14)]. A position paper of the European Association of Asthma, Allergy and Clinical Immunology (EAACI) outlined that it is of high importance to understand how diet diversity modulates allergic outcomes (15). The task force also recommends to use indices in the future to better describe the allergic potential of foods or food patterns within the context of diet diversity. There are several dietary indices available that have been analyzed with respect to nutritional quality, adherence to dietary guidelines or recommendations as well as in association with specific outcomes, such as cardiovascular disease risk (16, 17). Because allergies are characterized by inflammatory processes (18, 19), it would be appropriate to apply an index representing the inflammatory potential of the individual diet in this context (20, 21).

In addition to the role of specific dietary factors contributing to the pathogenesis of allergic and atopic conditions, it also is important to consider that individuals suffering from food allergen-triggered symptoms develop a specific dietary pattern due to their mandatory avoidance of causative allergens (7, 22–25). Such dietary restrictions are, themselves, known to be associated with adverse health issues (22). Knowing the dietary intake of individuals affected by allergies, in particular of children, might offer possibilities to improve their immune response, reduce symptom severity or relapse frequency which is even more important in growing individuals. Therefore, the aim of the present study was to evaluate the nutritional pattern in a cohort of 10-year-old children with respect to their development of allergic diseases known to be directly affected by dietary allergens such as atopic dermatitis or food allergy. Because of the role of inflammation in these conditions, the children’s Dietary Inflammatory Index (C-DII) was used to describe dietary exposure.

## Materials and Methods

### Study Design

Within the population-based, prospective birth cohort study LiNA (Lifestyle and environmental factors and their Influence on Newborn’s Allergy risk) 629 mother-child-pairs (622 mothers and 629 children; 7 twins) were recruited during regular appointments with their midwife during May 2006 and December 2008 in Leipzig, Germany. The aim of the study is to investigate how lifestyle and environmental factors in the pre and postnatal period influence the immune system of the newborn and the child later in life with consequences for future allergy risk. Mothers suffering from chronic immune or infectious diseases during pregnancy were excluded from the study, as well as mothers with non-German ancestry/non-Caucasian ethnicity. Further, only term (≥ 37th week of pregnancy, ≥ 2,500 g birth weight) and healthy newborns (without postnatal infections that needed medical treatment) were included. General characteristics (such as sex of the child, mothers age at birth, birth mode, breastfeeding duration, presence of older siblings, parental school education, environmental tobacco smoke (ETS) exposure, pet keeping, family history of atopy, etc.) or outcome data were assessed during pregnancy and annually thereafter using questionnaires and in-person examinations. All questionnaires were self-administered by the parents (together with the children when they were old enough). Study participation was voluntary and written informed consent was obtained of all participants. The LiNA study was approved by the Institutional Review Board of the University of Leipzig and the Saxonian Board of Physicians (046-2006, 160-2008, 160b/2008, 144-10-31052010, 113-11-18042011, 206-12-02072012, 169/13-ff, 150/14-ff, EK-allg-28/14-1, 008/17-ek).

### Dietary Assessment

Dietary intake was assessed at the age of 10 years using a semi-quantitative food frequency questionnaire (FFQ) asking for children’s intake of foods in the past 12 months. The FFQ contained 106 food items from 14 different food/beverage groups (bread and rolls, spreads, cheese and cold sausage, cereals and cornflakes, milk (-products) and eggs, basic carbohydrates, meat, fish, vegetables, fruits, cake and desserts, (salty) sweets and nuts, fats and oils and beverages) with nine non-overlapping frequency categories (never to ≥4 times/d) as well as five relative portion size options (1/4, 1/2, 1, 2, 3) referring to an exemplified or pictured standard portion size (i.e., Equal to 1). Relevant data on the fat content and type of preparation were recorded (e.g., fat content of milk products/raw or cooked vegetables etc.). The data based on FFQ where then analyzed using DGExpert (version 1.9, based on codes by the German Food Code and Nutrient Database – BLS 3.02) which outlined the individual intake of 158 macro- and micronutrients for every child. A comparison to applicable reference values (D (Germany) –A (Austria) –CH (Switzerland) reference) is provided by DGExpert considering the children’s personal data [age, sex, weight, height and Physical Activity Level (PAL)] to calculate individual energy- and nutrient requirements. Average PAL was considered to be 1.6 according to the German Society of Nutrition (DGE) and was further adopted according to children’s activity on their way to school (how children went to school (walk/bike/car) and how long this took). In addition to the nutrient calculation, the consumption of specific food groups was analyzed according to the optimized mixed diet (OptimiX) recommendation (beverages [mL/d], bread and cereals [g/d], pasta, rice and potatoes [g/d], vegetables [g/d], fruits [g/d], milk and dairy products [g/d], meat and sausages [g/d], eggs [pieces/week], fish [g/week], fats [g/d] or tolerated food group [portions/d, sweets, snacks, soft drinks] (26).

### Children’s Dietary Inflammatory Index

Using data from the FFQ the inflammatory potential of the participant’s diet was evaluated by calculating the children’s Dietary Inflammatory Index (C-DII) for each child. The detailed C-DII methodology has been established and described earlier (27). Briefly, the Dietary Inflammatory Index (DII) classifies human dietary patterns on a continuous scale from anti-inflammatory (values <0) to pro-inflammatory (values >0) based on a broad literature database with respect to 45 foods or nutrients that were described to be associated with inflammatory markers such as interleukin (IL)-1b, IL-4, IL-6, IL-10, tumor necrosis factor (TNF)-a and C-reactive protein (CRP) (28). The DII was further adapted for children (C-DII) using 25 nutrients or food parameters (27). All parameters except selenium (missing software database information) were included for the LiNA C-DII; Anti-inflammatory parameters included: vitamin A, thiamine (vitamin B1), riboflavin (vitamin B2), niacin (vitamin B3), vitamin B6, folic acid (vitamin B9), vitamin D, vitamin C, vitamin E, beta carotene, fiber, mono-unsaturated fatty acid (MUFA), poly-unsaturated fatty acid (PUFA), magnesium (Mg) and zinc (Zn); Pro-inflammatory parameters included: vitamin B12, energy, carbohydrates, total fat, saturated fat, cholesterol, protein, alcohol and iron (Fe). Next to data on the inflammatory potential of the diet, dietary intakes from a wide range of diverse populations from different countries representing six continents were used to construct a consumption database that was referred to as Z-Scores (27).

### Atopic Outcomes

Atopic dermatitis and food allergy were used as atopic outcomes in the present analyses. Atopic dermatitis was recorded annually *via* parental report of a doctor-diagnosed atopic dermatitis or as the diagnosis of the study physician at the annual LiNA medical examination. For food allergy the annual parental report of a doctor-diagnosed food allergy was used. Outcome prevalence was defined as at least one positive indication within the first 10 years of life.

### IgE Measurements

Total immunoglobulin E (IgE), as well as IgE specific for food allergens (fx5) or inhalative allergens (sx1) were determined at children’s age of 10 years by Phadia ImmunoCAP system (Thermo Fisher Scientific, Freiburg, Germany) from serum samples. Total IgE concentration >34.6 kU/l was classified as “increased,” as well as specific IgE (sx1 or fx5) >0.35 U/l (29). Values below the detection limit were included in the analyses using half of the defined detection limit.

### Statistical Analyses

After testing for normal distribution with Shapiro-Wilk test, descriptive analyses were performed using non-parametric tests for parameters found not to be distributed normally. Data are presented as medians with 25–75th percentile (1st to 3rd quartile) or as frequencies (%). χ^2^-tests were used to compare characteristics in the analyzed sub-cohort at age 10 years with the total cohort recruited during pregnancy (sex of the child, mothers age at birth, birth mode, breastfeeding duration, presence of older siblings, parental school education (highest level), environmental tobacco smoke (ETS) exposure during pregnancy, pet keeping during pregnancy, family history of atopy and body mass index). Further, these characteristics were compared within the cohort for analysis with respect to children’s anti-/pro-inflammatory C-DII. Characteristics known to be associated with atopic outcomes that were also associated with the C-DII were included as confounders in the regression models.

The relationship between atopic outcomes and nutrients/C-DII/food groups as well as the association between C-DII and food groups was addressed using the Mann–Whitney *U*-test. To adjust for confounders, multiple logistic regression models were used to calculate the risk of having a pro-inflammatory C-DII at the age of 10 years (dependent variable) with respect of the atopic outcome development within the first 10 years of life (independent variable) while adjusting for potential confounding factors (sex of the child, breastfeeding duration, parental school education, pet keeping during pregnancy and body mass index age 10). Data are presented as odds ratios with 95% confidence interval. All *p*-values <0.05 were considered to be significant. Statistical analyses were performed with STATISTICA for Windows, Version 13 (Statsoft Inc.), R (version 3.6.1; R development Core Team) or GraphPad Prism (Version 8.1.2.).

## Results

### Characteristics of the Analyzed LiNA Sub-Cohort

From the total cohort (*n* = 629), 268 participated in the 10-year campaign and 211 of whom were available with complete FFQ as well as confounding data (Supplementary Figure 1). Drop outs resulted from loss to follow-up over 10 years (average annual drop out 8.95%). Reasons for drop out – if available – were for example family moving or less available time when kids entered school. General characteristics (sex of the child, mothers age at birth, birth mode, breastfeeding duration, presence of older siblings, parental school education (highest level), environmental tobacco smoke (ETS) exposure during pregnancy, pet keeping during pregnancy, family history of atopy and body mass index) of the analyzed sub-cohort compared to the total LiNA cohort are presented in Table 1 with no differences seen between the two groups.

**TABLE 1:** Study characteristics.

	Analyzed sub-cohort Age 10 *N* = 211^a^ n (%)	Entire LiNA cohort Pregnancy *N* = 629^a^ n (%)	*p*-value χ^2^ test
**Sex of child**			0.80
Male	107 (50.7)	330 (52.5)	
Female	104 (49.3)	299 (47.5)	
**Mothers age at birth**			0.74
≤25 years	16 (7.58)	66 (10.5)	
>25 – 30 years	72 (34.1)	239 (38.0)	
>30 – 35 years	77 (36.5)	214 (34.0)	
>35	46 (21.8)	110 (17.5)	
**Birth mode**			0.72
Spontaneous	152 (72.0)	471 (74.9)	
Cesarean section	56 (26.5)	132 (21.0)	
Others	2 (1.00)	7 (1.10)	
**Breastfeeding duration**			0.56
No	11 (5.20)	26 (4.10)	
3 months	27 (12.8)	112 (17.8)	
6 months	60 (28.4)	190 (30.2)	
12 months	107 (50.7)	254 (40.4)	
**Presence of older siblings**			0.79
Yes	74 (35.1)	208 (33.1)	
No	136 (64.5)	414 (65.8)	
**Parental school education^b^**			0.64
Low	2 (1.00)	16 (2.50)	
Medium	43 (20.4)	142 (22.6)	
High	165 (78.2)	464 (73.8)	
**ETS^c^ exposure pregnancy**			0.34
No	168 (81.6)	464 (76.1)	
Yes	38 (18.4)	146 (23.9)	
**Pet keeping pregnancy**			0.62
No	128 (61.2)	358 (57.8)	
Yes	81 (38.8)	261 (42.2)	
**Family history of atopy**			0.97
None	69 (32.7)	212 (33.7)	
One	103 (48.8)	296 (47.1)	
Both	39 (18.5)	121 (19.2)	
**Body mass index age 10^d^**			
Underweight	25 (12.0)	–	
Normal weight	166 (79.4)	–	
Overweight/obese	18 (8.6)	–	

*^a^n may differ from 211/629 due to missing data.*

*^b^Low = 8 years school education; medium = 10 years school education; high = at least 12 years school education.*

*^c^Environmental tobacco smoke.*

*^d^Underweight (body mass index equivalent to <18,5 kg/m^2^ at 18 years), normal weight (body mass index equivalent to 18,5 −<25 kg/m^2^ at 18 years), overweight/obese (body mass index equivalent to ≥25 kg/m^2^ at 18 years). General characteristics of the analyzed sub-cohort compared to the total LiNA cohort.*

### Atopic Outcomes

Within the analyzed sub-cohort prevalence of atopic dermatitis and food allergy during the first 10 years of life was 37.4 and 11.8%, respectively (Supplementary Table 1). From the 79 children diagnosed with atopic dermatitis, 76.2% had increased total IgE levels measured at the age of 10 years compared to the allergy-diagnostic reference value of 34.6 kU/l for 10-year-old children. Further, 25.4% of these children had increased food-allergen-specific fx5 levels as well as 61.2% increased airway-allergen-specific sx1 levels. In addition, from the 25 children with food allergy 90.5% had increased total IgE levels, 33.3% increased fx5 levels as well as 71.4% increased sx1 levels at the age of 10 years. For 16 children both atopic dermatitis and food allergy was reported.

### General Dietary Intake

Because it was the first time that nutrients were assessed *via* FFQ and DGExpert in the LiNA cohort, a comparison of the final LiNA nutrient data set was performed with data from a study with similar design/geographical region as were available from EsKiMo, a nutritional module from the Robert Koch institute’s KiGGs study (30). All analyzed macronutrients (% of total energy intake for fat, carbohydrates and proteins, absolute amounts of fatty acids, cholesterol, sugar and fibers) or absolute amounts of consumed minerals or vitamins were in a very similar range and thus comparable between LiNA and EsKiMo (31) for 10-year-old boys and girls (Supplementary Table 2; overall median difference between LiNA and EsKiMo was 8%).

For the following investigations, a representative subset of 35 nutrients (macronutrients, minerals, and fat/water soluble vitamins) was analyzed. In general, the overall intake of macro- and micronutrients of the LiNA participants was displayed as % of total energy intake (carbohydrates, fat, and proteins) or as absolute values; both compared to the D-A-CH-reference values which is shown in Supplementary Figure 2 for all children and in Supplementary Figure 3 for boys/girls separately. For macronutrients in all children, data exceeded the recommendation for total fat intake (30% of energy) and total protein intake (0.9 g/kg body weight; in LiNA adequate to an overall 6.6% of the total energy intake) as pictured in Supplementary Figure 2A, with the girls being significantly lower in protein intake than the boys (Supplementary Figure 3A). Children’s minerals intake exceeded the D-A-CH reference for sodium (Na), chloride (Cl), magnesium (Mg), zinc (Zn), copper (Cu), and manganese (Mn), while calcium (Ca), phosphorus (P), iron (Fe) and iodine (I) and fluoride (F), in particular, were below D-A-CH reference values (Supplementary Figure 2B). According to sex differences, girls had a significant lower Na, Cl, K, Ca, P, Mg, Fe, I, F, and Cu intake than the boys (Supplementary Figure 3B). Potassium (K) intake was according to the recommendations. With respect to fat-soluble vitamins shown in Supplementary Figure 2C for the total sub-cohort, children were above (for vitamin A and K) and below (for vitamin E and in particular for vitamin D) the recommendation, with no differences between girls/boys (Supplementary Figure 3C). Water soluble vitamins (Supplementary Figure 2D) were all clearly on or above the recommended intake (for vitamin C, B1 (thiamine), B2 (riboflavin), B3 (niacin), B6, B7 (biotin), B9 (folate) and B12), with girls being significant lower in B5 (pantothenic acid), B7 and B12 intake than the boys (Supplementary Figure 3D). According to our data only vitamin B5 was consumed in amounts below the current recommendations (Supplementary Figure 2D), in particular by girls (Supplementary Figure 3D).

### Dietary Intake With Respect to Atopic Diseases

The dietary intake assessed at the age of 10 years was analyzed with respect to children’s development of atopic dermatitis or food allergy within the first 10 years of life (Table 2). Children with atopic dermatitis/food allergy consumed significantly lower amounts of fiber (in % of the total energy intake) than children without atopic dermatitis/food allergy. Sugar intake was lower in children with atopic dermatitis; however, overall sugar intake was above the recommendation of 10% of the total energy intake in all children. The intake of minerals was not different in children with or without atopic dermatitis/food allergy. Children with atopic dermatitis had a significant lower intake of vitamins C, E, and B7 compared to children who did not develop an atopic dermatitis within the first 10 years of life. However, both groups had either higher (for vitamin C and B7) or lower levels (for vitamin E) compared to the D-A-CH reference. Vitamin intake of children with food allergy was not different from those without food allergy within the first 10 years of life.

**TABLE 2:** Single nutrients and atopic outcomes.

	Atopic dermatitis within the first 10 years							Food allergy within the first 10 years						
	Without (*n* = 132)			With (*n* = 79)				Without (*n* = 186)			With (*n* = 25)			
	Median	Q 1st	Q 3rd	Median	Q 1st	Q 3rd	*p*-value	Median	Q 1st	Q 3rd	Median	Q 1st	Q 3rd	*p*-value #
**Macronutrients**														
Energy (kcal)	**2109**	1780	2590	**2152**	1715	2719	0.91	**2097**	1764	2608	**2464**	1826	2859	0.19
Fat (%E)	**35.5**	31.2	39.4	**36.4**	32.9	40.0	0.10	**35.8**	31.8	39.4	**36.3**	32.9	40.0	0.36
SFA (%E)	**15.3**	13.7	17.9	**16.0**	14.7	18.0	0.07	**15.7**	14.0	17.9	**15.8**	14.1	17.8	0.71
PUFAs (%E)	**4.7**	4.1	5.7	**4.7**	4.3	5.4	0.58	**4.7**	4.1	5.5	**4.6**	4.3	5.3	0.87
MUFAs (%E)	**12.3**	10.9	13.8	**12.6**	11.3	14.0	0.17	**12.3**	10.9	13.9	**12.8**	11.7	14.5	0.15
Chol (mg)	**286**	227	360	**293**	228	389	0.46	**283**	225	364	**330**	255	394	0.08
Carbs (%E)	**49.0**	45.5	53.5	**47.3**	44.3	51.6	0.09	**48.3**	44.5	53.1	**47.7**	44.4	52.0	0.53
Sugar (%E)	**21.9**	17.7	26.3	**19.5**	15.8	25.2	0.05	**20.6**	17.0	26.0	**20.6**	17.5	23.2	0.71
Fiber (%E)	**2.0**	1.7	2.5	**1.9**	1.5	2.2	0.04	**2.0**	1.6	2.4	**1.7**	1.5	2.0	0.02
Protein (%E)	**14.4**	13.2	15.8	**14.9**	13.3	16.2	0.23	**14.6**	13.2	16.1	**14.7**	13.8	15.5	0.91
Minerals*														
Na	**210.0**	166.4	280.5	**209.1**	162.7	298.2	0.67	**210.0**	165.5	286.4	**212.7**	186.4	300.9	0.39
K	**101.0**	79.0	121.0	**93.4**	73.8	117.2	0.23	**97.1**	75.9	120.7	**103.4**	84.8	111.0	0.75
Ca	**64.5**	53.6	89.5	**71.8**	50.9	91.8	0.92	**65.9**	51.8	88.2	**70.9**	55.5	91.8	0.64
Mg	**120.0**	96.5	151.3	**115.2**	90.0	154.8	0.36	**119.0**	95.7	151.3	**123.0**	96.0	153.2	0.69
P	**90.8**	74.4	116.8	**92.8**	72.8	119.2	0.91	**91.6**	72.8	115.2	**91.2**	77.6	124.0	0.48
Fe	**71.7**	56.0	88.9	**72.0**	54.2	94.2	0.90	**68.8**	55.0	90.0	**78.7**	56.0	97.5	0.33
Zn	**123.8**	94.8	144.3	**112.4**	93.7	155.0	0.71	**118.5**	93.7	147.0	**129.7**	102.4	144.6	0.83
J	**48.3**	38.6	60.3	**46.7**	38.3	61.1	0.48	**48.3**	38.3	60.0	**47.8**	38.3	66.7	0.96
Cl	**215.9**	173.8	293.2	**222.4**	161.2	305.3	0.89	**215.9**	170.6	288.8	**222.4**	183.5	315.9	0.42
Fl	**33.8**	27.8	42.8	**32.5**	26.5	44.0	0.66	**33.0**	27.0	42.5	**35.0**	30.0	45.5	0.30
Cu	**135.5**	109.5	165.0	**120.0**	98.0	180.0	0.20	**131.5**	106.0	168.0	**137.0**	109.0	169.0	0.85
Mn	**214.8**	163.3	286.3	**187.0**	152.5	281.5	0.21	**205.0**	157.5	284.5	**194.5**	161.5	281.0	0.84
Vitamins*														
A	**125.8**	93.8	172.9	**124.7**	95.7	176.2	0.94	**125.1**	94.0	175.4	**125.0**	98.1	161.9	0.92
C	**241.1**	164.8	327.8	**180.8**	125.7	344.3	0.02	**223.1**	145.4	327.5	**201.2**	135.2	352.5	0.84
D	**9.0**	7.0	13.0	**8.5**	5.5	13.5	0.59	**8.5**	6.5	13.0	**9.0**	5.5	13.5	0.66
E	**83.7**	68.8	114.3	**77.7**	60.0	103.6	0.03	**81.2**	63.6	108.2	**80.9**	66.2	110.0	0.94
K	**303.0**	210.4	417.4	**252.5**	175.3	400.5	0.14	**285.6**	197.8	411.5	**239.8**	182.3	396.5	0.68
B1	**146.9**	116.2	177.9	**140.0**	105.8	179.9	0.40	**143.8**	108.0	175.0	**156.0**	125.0	188.0	0.27
B2	**139.5**	109.5	169.5	**130.9**	101.8	169.1	0.43	**136.2**	106.0	167.0	**149.1**	120.9	178.2	0.41
B3	**240.5**	194.5	292.7	**230.9**	192.3	300.0	0.82	**236.7**	193.1	300.0	**260.8**	209.2	293.1	0.39
B5	**92.9**	74.8	116.8	**85.8**	66.8	109.4	0.11	**151.0**	118.0	194.0	**162.0**	131.0	203.0	0.48
B6	**157.0**	121.5	194.5	**150.0**	114.0	196.0	0.53	**223.5**	181.0	286.5	**252.5**	171.5	277.5	0.91
B7	**236.3**	188.3	285.3	**201.0**	158.5	285.5	0.047	**97.9**	75.8	135.0	**116.7**	72.9	133.3	0.85
B9	**105.6**	80.0	132.3	**88.8**	72.1	135.0	0.12	**216.0**	169.5	303.5	**270.5**	204.0	322.5	0.12
B12	**216.0**	162.3	301.8	**237.0**	177.5	319.5	0.24	**89.4**	73.2	113.6	**96.2**	76.0	108.4	0.67

*% of D-A-CH reference.

*# p-values from Mann–Whitney U-test, for medians with first/third quartile (Q 1st/Q 3rd).*

*%E - percentage of energy intake.*

*Median daily nutritional intake of 10-year old children with or without atopic dermatitis/food allergy within the first 10 years of life.*

*All Median values are printed in bold.*

### Children’s Dietary Inflammatory Index

**FIGURE 1:**
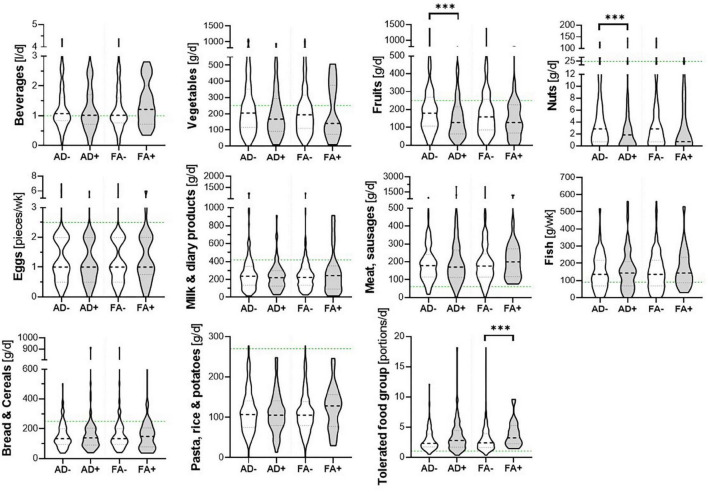
Food groups and atopic outcomes. Children’s consumed food groups at the age of 10 years according to having developed atopic dermatitis (AD +) or food allergy (FA +) within the first 10 years of life or not (AD- and FA-, respectively). Green line: OptiMix recommendations for 10–12 year old children. Data are presented as violin plots with median (bold dotted line) and 25 to 75th percentile (dotted line), *n* = 211. ***Significant difference (*p* < 0.05) between AD+/AD- or FA+/ FA-, respectively (Mann–Whitney *U*-test).

**FIGURE 2:**
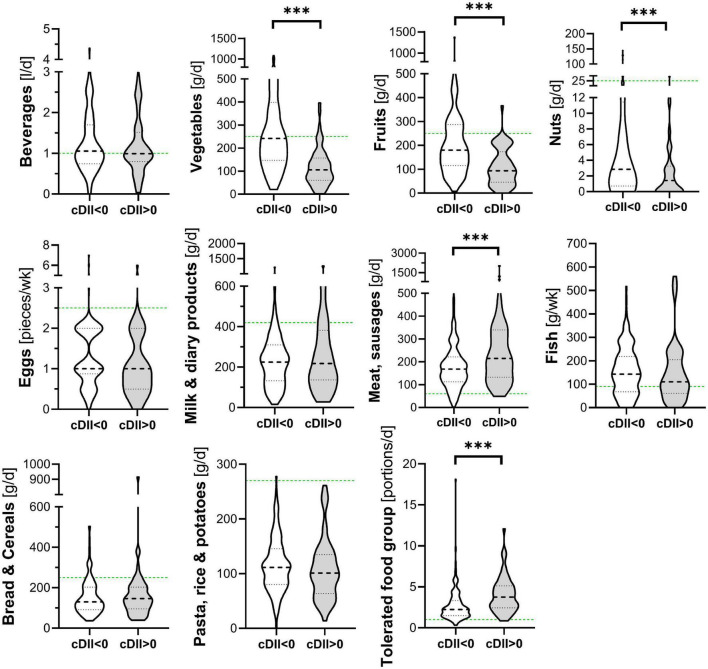
Food groups and C-DII. Children’s consumed food groups at the age of 10 years according to having a pro (C-DII >0) or anti-inflammatory (C-DII <0) diet at the age of 10 years. Green line: OptiMix recommendations for 10–12 year old children (26). Data are presented as violin plots with median (bold dotted line) and 25 to 75th percentile (dotted line), *n* = 211. ^***^Significant difference (*p* < 0.05) by Mann–Whitney *U*-test.

Children’s dietary inflammatory index scores were calculated to quantify the inflammatory potential of the diet of LiNA children. In general, values above 0 indicate a more pro-inflammatory pattern, whereas values below 0 indicate an anti-inflammatory pattern. Overall, the LiNA children had a median C-DII of −0.97 (interquartile range (IQR): −2.06 to 0.26; *n* = 211), with girls being lower than boys (i.e., −1.22 (IQR: −2.17 to −0.24; *n* = 104 compared to −0.53 (IQR: −1.93 to 0.77; *n* = 107), respectively. Furthermore, general characteristics were compared between children who had a more anti-inflammatory (C-DII <0) and those who had a pro-inflammatory (C-DII >0) dietary pattern (Table 3). Data revealed that sex, breastfeeding duration, parental school education and children’s body mass index at the age of 10 years differed between children with pro-inflammatory and those with anti-inflammatory C-DII score. Next, the C-DII was analyzed in the context of atopic dermatitis and food allergy. As shown in Table 4, there were no significant differences in C-DII between children with and those without atopic outcomes, although C-DII levels tended to be lower (indicating a more anti-inflammatory diet) in children without atopic outcomes. When C-DII was grouped into anti-inflammatory (<0) and pro-inflammatory (>0), regression models revealed that having atopic outcomes was associated with having a pro-inflammatory pattern at the age of 10 years (Table 5; OR = 2.22, 95% CI: 1.14–4.31) for children with atopic dermatitis, OR = 3.82 (95% CI: 1.47–9.93) for children with food allergy in the first 10 years of life). These associations were independent of confounders. This more pro-inflammatory pattern in children with atopic outcomes was supported by analyses of specific consumed food groups: children that developed atopic dermatitis within the first 10 years of life consumed significantly less fruits and nuts, children with food allergy consumed significantly more of the tolerated food group including sweets/snacks etc. (Figure 1). Children with a pro-inflammatory diet (C-DII >0) consumed fewer vegetables, fruits and nuts, but more meat/sausages and more sweets/snacks (Figure 2).

**TABLE 3:** C-DII and study characteristics.

	C-DII class 1 Anti-inflammatory (*n* = 151^a^)		C-DII class 2 Pro-inflammatory (*n* = 60^a^)		*p*-value
	n	%	n	%	χ^2^ test
**Sex of the child**					
Male	70	46.4	37	61.7	0.02
Female	81	53.6	23	38.3	
**Mothers age at birth**					
≤25 years	13	8.6	3	5.0	0.45
>25 – 30 years	48	31.8	24	40.0	
>30 – 35 years	58	38.4	19	31.7	
>35	32	21.2	14	23.3	
**Birth mode**					
Spontaneous	108	72.0	44	73.3	0.52
C. section	40	26.7	16	26.7	
Others	2	1.3	0	0	
**Breastfeeding duration**					
no	10	6.8	0	0	0.047
3 month	18	12.2	9	15.8	
6 month	41	27.7	19	33.3	
12 months	79	53.4	28	49.1	
**Presence of older siblings**					
Yes	52	34.7	22	36.7	0.77
No	98	65.3	38	63.3	
**Parental school education^b^**					
Low	2	1.3	0	0	0.049
Medium	25	16.7	18	30	
High	123	82.0	42	70	
**ETS exposure pregnancy**					
No	122	83.6	46	76.7	0.22
Yes	24	16.4	14	23.3	
**Pet keeping during pregnancy**					
No	97	65.1	31	51.7	0.06
Yes	52	34.9	29	48.3	
**Family history of atopy**					
None	47	31.1	22	36.7	0.19
One	72	47.7	31	51.7	
Both	32	21.2	7	11.7	
**Body mass index age 10^c^**					
Under weight	17	11.4	8	13.3	0.02
Normal weight	115	77.2	51	85	
Overweight/obese	17	11.4	1	1.7	

*^a^ n may differ from 151/60 due to missing data.*

*^b^ Low = 8 years school education; medium = 10 years school education; high = at least 12 years school education.*

*^c^ Underweight (body mass index equivalent to 18,5 kg/m^2^ at 18 years), normal weight (body mass index equivalent to 18,5 −<25 kg/m^2^ at 18 years), overweight/obese (body mass index equivalent to ≥25 kg/m^2^ at 18 years). ETS - environmental tobacco smoke. General characteristics of the analyzed sub-cohort with respect of having an anti-inflammatory (C-DII <0) or pro-inflammatory (C-DII >0) children’s dietary inflammatory index at the age of 10 years.*

**TABLE 4:** C-DII and atopic outcomes.

Atopic dermatitis within the first 10 years									
	**Without disease outcome**				**With disease outcome**				
	**n**	**Median**	**1st quartile**	**3rd quartile**	**n**	**Median**	**1st quartile**	**3rd quartile**	***p*-value^#^**
All	132	**−1.19**	−2.13	−0.06	79	**−0.53**	−2.00	0.79	0.05
Boys	60	**−0.97**	−2.07	0.17	47	**−0.27**	−1.58	1.07	0.12
Girls	72	**−1.26**	−2.15	−0.32	32	**−1.19**	−2.18	0.40	0.57

**Food allergy within the first 10 years**									
	**Without disease outcome**				**With disease outcome**				
	**n**	**Median**	**1st quartile**	**3rd quartile**	**n**	**Median**	**1st quartile**	**3rd quartile**	***p*-value**

All	186	**−1.07**	−2.13	0.07	25	**−0.25**	−1.79	0.73	0.08
Boys	92	**−0.62**	−1.98	0.75	15	**−0.25**	−1.79	1.07	0.42
Girls	94	**−1.31**	−2.18	−0.35	10	**−0.03**	−2.06	0.73	0.12

*# p-values from Mann-Whitney U-test, for medians with first/third quartile. Descriptive data on the children’s dietary inflammatory index (C-DII) assessed at the age of 10 with respect to their development of atopic dermatitis or food allergy within the first 10 years of life. Shown among all 211 children from the LiNA-cohort, as well as separately for boys and girls, p-value from Mann-Whitney-U-test.*

**TABLE 5:** Pro-inflammatory C-DII and atopic outcomes.

	Pro-inflammatory diet age 10 (C-DII >0)											
AD		Crude						Adjusted*				
	n total	n C-DII >0	OR	(95% CI)		*p*-value	n total	n C-DII >0	OR	(95% CI)		*p*-value
All	211	60	1.89	1.02	3.49	0.04	201	57	2.22	1.14	4.31	0.02
Boys	107	37	1.87	0.83	4.23	0.13	102	36	2.65	1.04	6.72	0.04
Girls	104	23	1.62	0.61	4.31	0.33	99	21	2.52	0.86	7.36	0.09

	**Pro-inflammatory diet age 10 (C-DII >0)**											
**FA**		**Crude**						Adjusted*				
	**n total**	**n C-DII >0**	**OR**	**(95% CI)**		***p*-value**	**n total**	**n C-DII >0**	**OR**	**(95% CI)**		***p*-value**

All	211	60	2.65	1.13	6.24	0.03	201	57	3.82	1.47	9.93	0.01
Boys	107	37	1.81	0.59	5.53	0.29	102	36	4.57	1.17	17.9	0.03
Girls	104	23	4.22	1.09	16.4	0.04	99	21	7.24	1.51	34.8	0.01

*Logistic regression model adjusted for sex, breastfeeding duration, parental school education, pet keeping pregnancy and body mass index age 10; 10 missing cases on specific confounders, OR - odds ratio, CI - confidence interval. Logistic regression models – raw or adjusted for confounders - showing the risk for children consuming a pro-inflammatory diet at the age of 10 years (by having a C-DII >0) with respect to having developed atopic dermatitis (AD) or food allergy (FA) within the first 10 years of life.

## Discussion

In this project we assessed the dietary intake of 10-year-old children for the first time within the prospective birth cohort, LiNA. According to participants’ overall dietary intake, we were able to show that they had an adequate intake of the majority of nutrients, with some even exceeding the recommendations (e.g., for total fat, SFA, protein, sugar, Na and Cl). In contrast, for some nutrients children did not even reach half of the recommendation (e.g., vitamin D, F, I). When compared to other studies, LiNA results on the intake of specific nutrients, as well as the consumed food groups [according to the OptiMix recommendation (26)] were very similar to other studies; for example, compared to the nutritional assessment within Robert-Koch-Institute’s EsKiMo module (31). The overall median nutrient difference between children from LiNA and EsKiMo was 8%, supported by similar data on food consumption with respect to the recommendation as shown for 6–11 year-old children from EsKiMo: lower consumption of vegetables, fruits and carbohydrates (bread, cereals, pasta, rice and potatoes) as well as higher consumption of meat (meat, sausages, etc.) and sweets and snacks (31). Further, our data indicated that girls’ intakes of minerals and water soluble vitamins was higher than boys’ intakes. Sex-specific differences in food groups that provide these nutrients such as vegetables were also described previously (32–34).

In addition to the overall dietary pattern of the LiNA children, analyses on the intake of single nutrients and atopic outcomes were performed. We were not able to show a clear allergy-specific dietary pattern, although there were some changes in vitamins (B7, C, and E), sugar and fiber. Still, when interpreted according to the D-A-CH-references, these nutrients were lower (vitamin E) or higher (vitamin B7 and C and sugar) than the recommendation values independent of children’s allergy development. However, children with atopic dermatitis/food allergy within the last 10 years of life were more likely to show a less anti-inflammatory/more pro-inflammatory dietary pattern at the age of 10 years as assessed *via* the C-DII. In line with this, we were also able to show that children who developed atopic dermatitis within the first 10 years of life consumed significantly less fruits and nuts – food groups which, next to others, provide mostly nutrients that would drive the C-DII toward an anti-inflammatory pattern. This was supported by the significantly lower intake of vitamin C, E, and B7 in LiNA children with atopic dermatitis within the first 10 years of life. In addition, children with food allergy within the first 10 years of life consumed significantly more of the tolerated food group [which emphasizes sweets/snacks etc., according to Kersting et al. (26)]. This food group provides mostly nutrients that drive the C-DII toward a pro-inflammatory pattern such as sugar or saturated fat (with the consequence that these energy-dense foods result in higher overall energy intake and greater inflammation). This was confirmed by showing that children’s pro-inflammatory diet was associated with a lower intake of vegetables, fruits and nuts and a higher intake of meat products and sweets/snacks. We hypothesize that children might have developed this less-anti-inflammatory/more pro-inflammatory diet due to an avoidance of possible anti-inflammatory – but allergy triggering – food items. It was described earlier that therapeutic strategies in atopic dermatitis and food allergy often involve dietary exclusions, which may be seen as mandatory in children sensitive to food allergens for whom accidental and potentially life threatening anaphylactic reactions can occur (6). It was also described that these exclusions may impact diet quality, nutrient intake and nutrient demands. It was even shown that an unsupervised elimination diet in childhood might lead to malnutrition, growth retardation, vitamin deficiencies and associated health issues (22, 35). In the context of this study, a pro-inflammatory diet consumed by the children might worsen the atopic outcome itself (20) and furthermore reduce their buffering capacity against harmful environmental exposures or triggers. An optimal nutritional status was described to be protective against both communicable and non-communicable diseases (36).

Because of the design of this study, reverse causality could not be ruled out. The diet as assessed at the age of 10 years could be a proxy for children’s lifelong dietary pattern. The C-DII assessed in LiNA at the age of 10 was associated with markers of socio-economic status (SES) of their families assessed during pregnancy (such as parental school education). It has been shown previously that lower SES is associated with poorer nutrition (37). Studies also suggest that children begin to assimilate and mimic their parents’ food choices at a very young age (38) and that this parent-child-transmission in dietary behaviors is dependent on SES (39). It was further shown that parental SES impacts childhood health issues (40) and that healthy lifestyle promotion alone might not substantially reduce the socioeconomic inequity in health (41). So, it also should be kept in mind that a pro-inflammatory diet consumed by the children on a daily basis throughout infancy also might have contributed to their allergy development. However, with the data available in our cohort, we are not able to examine the direction of temporal ordering of these effects in further detail.

To the best of our knowledge, this is the first use of the C-DII in association with atopic dermatitis and food allergy. Both atopic dermatitis and food allergy are characterized by inflammatory processes (18, 19), similar to other non-communicable diseases that are characterized by low-grade, chronic systemic inflammation. It was shown, for example, that in adults a pro-inflammatory diet (DII >0) was associated with an increased risk of certain cancers, cardiovascular disease, adverse mental health outcomes, and musculoskeletal disorders (21). It also is known that a pro-inflammatory diet is linked to greater all-cause mortality risk (42). The evidence for an association between DII and respiratory health, neurodevelopmental outcomes, metabolic syndrome, diabetes and obesity was described to be either conflicting or scarce (21). Furthermore, there are limited data in the context of DII and allergies, and the available data so far address mainly respiratory issues such as asthma or wheezing. For example, in children, a pro-inflammatory diet was not associated with current asthma or lung function, but in children with allergic airway inflammation, a higher DII score was associated with a 2.38 fold higher risk of wheezing (43). In addition, a pro-inflammatory diet was associated with asthma (20). Further, it was shown that higher inflammatory potential of the maternal diet was associated with increased odds of offspring asthma and/or wheeze by age 4 years, although results attenuated into non-significance after adjustment for confounders (44).

One strength of our study lies in the well-characterized participants regarding longitudinal atopic outcomes and exposure variable assessment, including diet. Therefore, a possible link between children’s dietary intake of specific nutrients or specific indices such as the C-DII and allergy development could be investigated. The use of an index such as the C-DII offers an insight into the total dietary pattern compared to interpreting singular effects of specific nutrients. A limitation of the LiNA study in general is the potential bias by high rates of participating atopic parents (64.7%), limiting our ability to extrapolate findings to the general population (with approximately 30% prevalence of atopic outcomes). This fact is accumulating even more throughout the 10-year follow up, in detail 76% of the children positive for AD within our analyzed sub-cohort show a positive family history of atopy. This shift was also seen in the high rates of increased IgE levels in children negative for atopic dermatitis or food allergy. One further limitation of the study is the low number of cases in certain outcomes, in particular when analyses are stratified for sex, which limited the power of the results. Furthermore, outcome data were obtained, in part, from parental questionnaire documented physician diagnosis of outcomes. This might reduce the strengths of the reported results. However, by including clinical allergy markers such as the IgE data we may overcome this limitation, at least in part. Further, the high rates of increased IgE levels at the age of 10 years in children positive of atopic dermatitis or food allergy at least once during their first 10 years outlines that these children have a persistent atopic phenotype also at the age of 10 years. Another major limitation is the missing questionnaire information on children’s physical activity in general and in their leisure time in particular. Therefore, we probably have under-estimated the energy expenditure in several children. However, our data are very similar to the study protocol from Eskimo (30) who also reported this limitation.

## Conclusion

Children with atopic dermatitis/food allergy within their first 10 years of life were more likely to show a more pro-inflammatory dietary pattern assessed at the age of 10 years *via* the C-DII compared to children without allergic diseases. Because of their allergy history, these children may have developed a more pro-inflammatory dietary pattern due to avoidance of possible allergy triggers such as fruits or nuts for example. Overall, a pro-inflammatory dietary pattern might worsen the atopic outcome itself and reduce the buffering capacity of the individual against harmful environmental exposures or triggers. For pediatricians it is recommended to test affected children for their individual tolerance of allergenic foods to avoid a restrict elimination diet. Furthermore, an increased nutrient density of tolerable food items should be advised to omit undesirable effects of eating a pro-inflammatory diet.

## Data Availability Statement

The datasets presented in this article are not readily available because longitudinal LiNA datasets are not anonymized. Therefore, the raw cohort data cannot be provided as an open source file due to ethical declaration/data protection issues. Data can be requested in their analysed version from the corresponding author. Requests to access the datasets should be directed to KJ, kristin.junge@ufz.de.

## Ethics Statement

The studies involving human participants were reviewed and approved by the Institutional Review Board of the University of Leipzig and the Saxonian Board of Physicians (046-2006, 160-2008, 160b/2008, 144-10-31052010, 113-11-18042011, 206-12-02072012, 169/13-ff, 150/14-ff, EK-allg-28/14-1, and 008/17-ek). Written informed consent to participate in this study was provided by the participants’ legal guardian/next of kin.

## Author Contributions

KJ: conceptualization and project administration. OS, LB, NS, JH, and JF: methodology. OS, LB, and NS: software. OS, KJ, and SR: validation. OS and KJ: formal analysis, visualization, and writing – original draft preparation. AZ and GH: resources. MB, US, and WK: clinical resources. SR: data curation. GS, GH, JF, JH, AZ, OS, LB, SR, MB, US, NS, and WK: writing – review and editing. KJ and GS: supervision. GH: cohort PI. All authors contributed to the article and approved the submitted version.

## Conflict of Interest

JH owns controlling interest in Connecting Health Innovations LLC (CHI), a company that has licensed the right to his invention of the dietary inflammatory index (DII^®^) from the University of South Carolina in order to develop computer and smart phone applications for patient counseling and dietary intervention in clinical ettings. NS is an employee of CHI. The remaining authors declare that the research was conducted in the absence of any commercial or financial relationships that could be construed as a potential conflict of interest.

## Publisher’s Note

All claims expressed in this article are solely those of the authors and do not necessarily represent those of their affiliated organizations, or those of the publisher, the editors and the reviewers. Any product that may be evaluated in this article, or claim that may be made by its manufacturer, is not guaranteed or endorsed by the publisher.

## Abbreviations

AD: atopic dermatitis
C-DII: children’s Dietary Inflammatory Index
FA: food allergy
fx5: specific IgE for food allergen (mix)
IL: interleukin
sx1: specific IgE for respiratory allergen (mix)
OR: odds ratio.
