# Supplementary material for: Pro-inflammatory Diet Pictured in Children With Atopic Dermatitis or Food Allergy: Nutritional Data of the LiNA Cohort
Source: Front Nutr. 2022 Apr 8;9:868872. doi: 10.3389/fnut.2022.868872 (PMC9024336; doi:10.3389/fnut.2022.868872)
Supplement: Supplementary file 1 [file Presentation_1.pdf]

SUPPLEMENTARY MATERIAL

Original article

Pro-inflammatory diet pictured in children with atopic dermatitis or food allergy: nutritional data of the LiNA cohort

Olivia Schütte[1], Larissa Bachmann[1], Nitin Shivappa[2,3,4], James R. Hebert[2,3,4], Janine F. Felix[5,6], Stefan Röder[1], Ulrich Sack[7], Michael Borte[8], Wieland Kiess[9], Ana C. Zenclussen[1,10] Gabriele I. Stangl[11,*], Gunda Herberth[1,*], Kristin M. Junge[1,11,*]

1 Department of Environmental Immunology, Helmholtz Centre for Environmental Research – UFZ, Leipzig, Germany
2 Cancer Prevention and Control Program, University of South Carolina, Columbia, SC 29208, USA
3 Department of Epidemiology and Biostatistics, Arnold School of Public Health, University of South Carolina, Columbia, SC 29208, USA
4 Department of Nutrition, Connecting Health Innovations LLC, Columbia, SC 29201, USA
5 The Generation R Study Group (Na-2918), Erasmus MC, University Medical Centre Rotterdam, Rotterdam, Netherlands
6 Department of Pediatrics, Erasmus MC, University Medical Centre Rotterdam, Rotterdam, The Netherlands
7 Institute of Clinical Immunology, Medical Faculty, University of Leipzig, Leipzig, Germany
8 Children's Hospital, Municipal Hospital "St. Georg", Leipzig, Germany
9 Centre for Pediatric Research Leipzig, University Hospital for Children & Adolescents, Medical Faculty, University of Leipzig, Germany
10 Perinatal Immunology, Saxonian Incubator for Clinical Translation (SIKT), Medical Faculty, University of Leipzig, Germany
11 Institute for Agricultural and Nutritional Sciences, Martin Luther University Halle-Wittenberg, Halle/Saale, Germany

*shared authorship

Corresponding author:
Dr. Kristin Junge, Helmholtz-Centre for Environmental Research – UFZ, Department of Environmental Immunology, Permoserstrasse 15, 04318 Leipzig, phone: 0049 341 235 1550, mail: kristin.junge@ufz.de

Supplementary Table S1: Atopic outcome characterisation. Prevalence of atopic dermatitis and food allergy within the first 10 years of life within the analysed LiNA sub-cohort (n=211) and IgE levels measured in serum samples at the age of 10 years

	**Total cases**<br><br><br>**n (%)**	**tIgE** [e]<br>increased vs not increased<br>**n (%)**	**fx5** [f]<br>increased vs not increased<br>**n (%)**	**sx1** [f]<br>increased vs not increased<br>**n (%)**
**Atopic dermatitis**				
positive	79 (37.4) [a]	51 (76.2) / 16 (23.9)	17 (25.4) / 50 (74.6)	41 (61.2) / 26 (38.8)
negative	132 (62.6) [b]	65 (58.6) / 46 (41.4)	40 (36.0) / 71 (64.0)	11 (9.9) / 100 (90.1)
**Food allergy**				
positive	25 (11.8) [c]	19 (90.5) / 2 (9.5)	7 (33.3) / 14 (66.7)	15 (71.4) / 6 (28.6)
negative	186 (88.2) [d]	97 (61.8) / 60 (38.2)	21 (13.4) / 136 (86.6)	66 (42.0) / 91 (58.0)

[a,b,c,d] 33 cases with missing IgE measurements at the age of 10 years, in detail [a] 12 [b] 21 [c] 4 [d] 29
[e] increased: total IgE > 34.6 kU/L; [f] increased: specific IgE > 0.35 kU/L (CAP-class > 0)

Supplementary Table S2: Nutritional data validation. Comparison of average daily nutritional intake (absolute values) in 10 year old children from the LiNA- and EsKiMo cohort. Shown are median with 5. and 95. Percentile. Energy % - percentage of energy intake, RE – retinol equivalents, FE – folate equivalents
(EsKiMo data from Mensink, G., Heseker, H., Richter, A., et al. (2007). *Forschungsbericht Ernährungsstudie als KIGGS-Modul (EsKiMo)*.)

		**Girls**						**Boys**					
		**LiNA (n = 104)**			**EsKiMo (n = 93)**			**LiNA (n = 107)**			**EsKiMo (n = 99)**		
		**Median**	5. percentile	95. percentile	**Median**	5. percentile	95. percentile	**Median**	5. percentile	95. percentile	**Median**	5. percentile	95. percentile
	**energy (kcal)**	**1954.0**	1250.5	3084.6	**1842.4**	1234.2	2443.8	**2254.8**	1267.5	3498.8	**1813.3**	1296.9	2681.9
	water (l)	**1.7**	1.1	3.5	**1.6**	1.0	2.7	**2.1**	1.1	3.8	**1.7**	1.1	2.5
**Macronutrients**	fat (energy %)	**35.3**	25.7	45.1	**32.2**	21.7	41.4	**36.0**	24.6	43.9	**32.3**	22.9	41.6
	saturated FA (g)	**33.4**	18.5	60.7	**28.4**	12.8	42.8	**39.0**	22.7	77.0	**28.3**	17.6	49.2
	polyunsaturated FA (g)	**10.9**	5.9	20.8	**8.3**	3.6	17.9	**12.2**	7.6	22.4	**8.3**	4.6	15.1
	monounsaturated. FA (g)	**27.2**	14.9	48.5	**22.7**	10.2	38.0	**31.9**	18.2	59.9	**23.4**	14.3	39.5
	cholesterol (mg)	**271.5**	159.0	443.0	**249.3**	94.2	459.0	**305.0**	166.0	585.0	**244.9**	101.0	493.0
	carbohydrates (energy %)	**48.6**	39.8	59.4	**52.7**	43.6	66.4	**47.9**	39.7	60.4	**52.8**	43.0	64.5
	sugar (g)	**106.5**	50.1	177.0	**110.7**	55.6	193.8	**115.0**	46.6	235.0	**117.2**	66.3	208.5
	fibre (g)	**21.3**	11.0	38.9	**17.1**	9.3	27.2	**22.5**	11.9	47.7	**17.4**	9.0	28.8
	protein (energy %)	**14.7**	11.7	17.7	**13.1**	10.3	18.0	**14.6**	11.6	18.4	**13.7**	10.3	18.1
**Minerals**	sodium (g)	**2.2**	1.2	4.4	**2.2**	1.3	3.7	**2.5**	1.3	4.4	**2.2**	1.3	3.8
	potassium (g)	**2.7**	1.5	4.4	**2.3**	1.1	3.4	**3.1**	1.6	4.7	**2.3**	1.5	3.4
	calcium (mg)	**655.0**	380.0	1430.0	**871.2**	401.8	1.428.7	**840.0**	390.0	1790.0	**908.0**	476.2	1.424.9
	magnesium (mg)	**266.0**	148.0	454.0	**294.1**	151.4	460.6	**321.0**	167.0	509.0	**285.9**	186.4	432.1
	phosphorus (mg)	**1065.0**	680.0	2010.0	**1068.7**	531.3	1529.0	**1270.0**	690.0	2240.0	**1097.7**	726.4	1653.4
	iron (mg)	**9.2**	5.2	15.3	**10.4**	6.7	16.4	**10.3**	6.1	15.8	**11.0**	7.2	19.2
	zinc (mg)	**8.9**	5.7	15.8	**8.3**	4.7	12.6	**10.0**	5.9	18.2	**8.7**	6.0	13.2
	iodine (µg)	**83.5**	45.0	125.0	**77.6**	33.7	156.0	**96.0**	50.0	171.0	**79.1**	48.3	172.4
**Vitamins**	vitamin A (RE (mg))	**1.1**	0.6	3.2	**0.7**	0.2	2.2	**1.1**	0.5	3.1	**0.9**	0.4	2.1
	vitamin C (mg)	**148.3**	60.8	343.6	**100.0**	30.8	213.2	**139.4**	42.9	345.7	**99.2**	27.6	256.5
	vitamin D (µg)	**1.7**	0.9	4.3	**1.4**	0.2	3.9	**1.9**	0.9	4.7	**1.5**	0.5	6.6
	vitamin E (mg)	**9.1**	5.2	21.9	**9.7**	4.5	20.9	**10.3**	5.0	22.3	**9.2**	5.4	19.6
	vitamin K (µg)	**107.7**	42.0	274.0	**190.9**	93.7	395.7	**116.1**	38.5	266.6	**185.4**	97.6	320.2
	thiamine (mg)	**1.2**	0.7	2.3	**1.2**	0.6	2.3	**1.5**	0.8	2.3	**1.2**	0.7	2.6
	riboflavin (mg)	**1.3**	0.8	2.0	**1.5**	0.8	2.4	**1.6**	0.9	2.8	**1.5**	0.9	3.3
	niacin equivalents (mg)	**27.0**	17.7	42.8	**21.7**	12.9	29.7	**29.8**	18.8	48.6	**22.0**	14.4	38.8
	pyridoxine (mg)	**1.4**	0.8	2.5	**1.5**	0.8	2.4	**1.6**	0.8	2.7	**1.5**	0.9	3.8
	biotin (µg)	**42.5**	22.4	73.4	**37.0**	11.3	95.6	**50.5**	23.3	84.5	**39.4**	23.2	132.9
	folic acid (FE (µg))	**229.5**	136.0	409.0	**203.5**	115.8	387.2	**259.0**	233.0	446.0	**204.9**	105.4	495.7
	cyanocobalamin (µg)	**4.1**	2.4	7.8	**3.6**	1.3	6.8	**5.0**	2.3	9.9	**4.2**	2.0	8.7

Supplementary Figure Legends

Supplementary Figure S1: Flow chart. Included study subjects in the current project and reasons for drop outs/missing data.

Supplementary Figure S2: Single nutrient intake in all children. Nutrient intake as % of the D-A-CH reference values (log10). Data are presented in violin plots as median (solid line) with 25th to 75th percentile (dotted line), n = 211. A: Macronutrient, B: Water soluble vitamins, C: Fat soluble vitamins, D: Minerals. CH: carbohydrates, Na: sodium, Cl: chloride, K: potassium, Ca: calcium, P: phosphorus, Mg: magnesium, Fe: iron, I: iodine, F: fluoride, Zn: zinc, Cu: copper, Mn: manganese, vit – vitamin

Supplementary Figure S3: Single nutrient intake in girls and boys. Sex – specific nutrient intake as % of the D-A-CH reference values (log10). Data are presented in violin plots as median (solid line) with 25th to 75th percentile (dotted line) for boys (green, n = 107) and for girls (orange, n = 104), *** significant difference ($p < 0.05$) between girls and boys (Mann-Whitney U-test) A: Macronutrient, B: Water soluble vitamins, C: Fat soluble vitamins, D: Minerals. CH: carbohydrates, Na: sodium, Cl: chloride, K: potassium, Ca: calcium, P: phosphorus, Mg: magnesium, Fe: iron, I: iodine, F: fluoride, Zn: zinc, Cu: copper, Mn: manganese, vit – vitamin
