# Supplementary figures and images for: Pro-inflammatory Diet Pictured in Children With Atopic Dermatitis or Food Allergy: Nutritional Data of the LiNA Cohort
Source: Front Nutr. 2022 Apr 8;9:868872. doi: 10.3389/fnut.2022.868872 (PMC9024336; doi:10.3389/fnut.2022.868872)

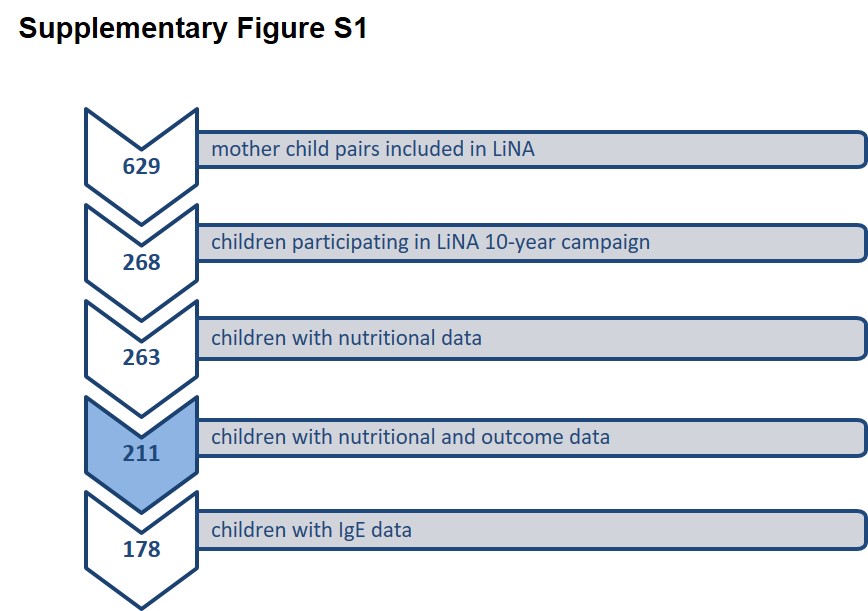

Supplement: Supplementary file 2 [file Image_1.JPEG]

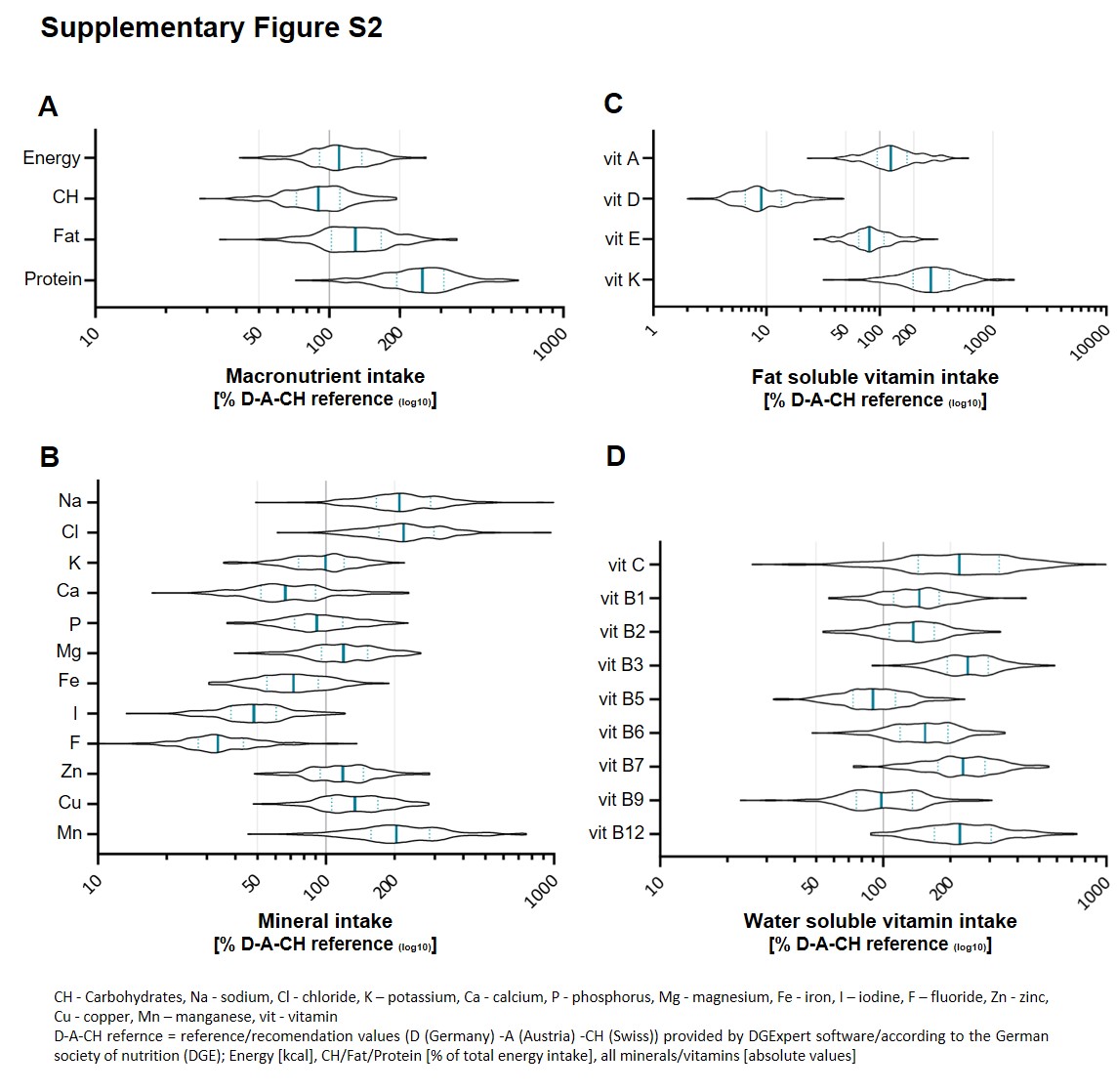

Supplement: Supplementary file 3 [file Image_2.JPEG]

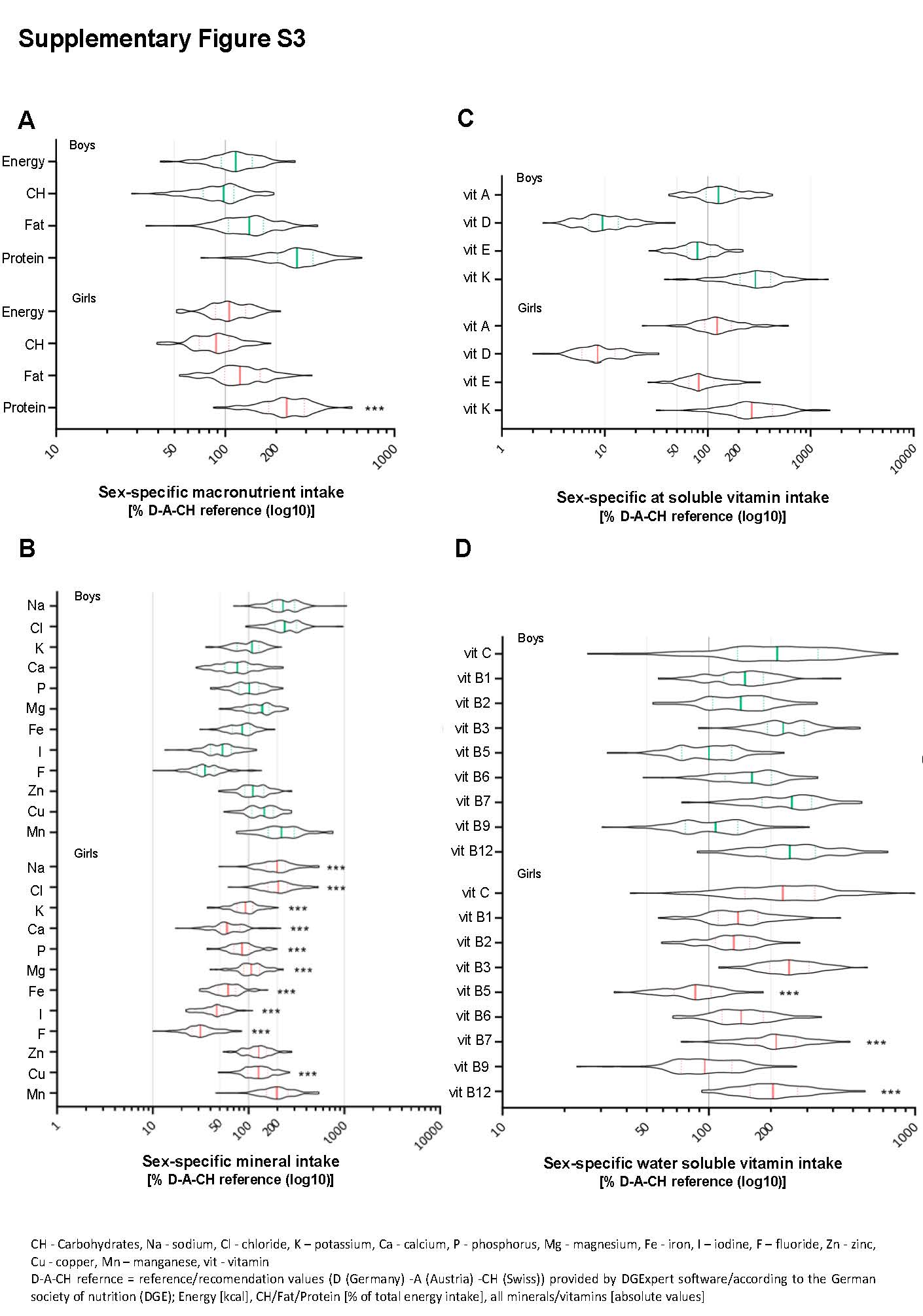

Supplement: Supplementary file 4 [file Image_3.jpg]
